# Medical resources and coronavirus disease (COVID-19) mortality rate: Evidence and implications from Hubei province in China

**DOI:** 10.1371/journal.pone.0244867

**Published:** 2021-01-15

**Authors:** Lin Xie, Hualei Yang, Xiaodong Zheng, Yuanyang Wu, Xueyu Lin, Zheng Shen

**Affiliations:** 1 Institution of Population and Labor Economics, The Chinese Academy of Social Science, Beijing, China; 2 School of Public Administration, Zhongnan University of Economics and Law, Wuhan, China; 3 School of Economics, Zhejiang Gongshang University, Hangzhou, China; 4 School of Economics and Management, Zhejiang A&F University, Hangzhou, China; National Institute for Infectious Diseases Lazzaro Spallanzani-IRCCS, ITALY

## Abstract

In light of the ongoing coronavirus disease (COVID-19) pandemic, this study aims to examine the relationship between the availability of public health resources and the mortality rate of this disease. We conducted empirical analyses using linear regression, a time-varying effect model, and a regression discontinuity design to investigate the association of medical resources with the mortality rate of the COVID-19 patients in Hubei, China. The results showed that the numbers of hospital beds, healthcare system beds, and medical staff per confirmed cases all had significant negative effects on the coronavirus disease mortality rate. Furthermore, in the context of the severe pandemic currently being experienced worldwide, the present study summarized the experience and implications in pandemic prevention and control in Hubei province from the perspective of medical resource integration as follows: First, hospitals’ internal medical resources were integrated, breaking interdepartmental barriers. Second, joint pandemic control was realized by integrating regional healthcare system resources. Finally, an external medical resource allocation system was developed.

## Introduction

The coronavirus disease (COVID-19) pandemic that unexpectedly emerged toward the end of 2019 has attracted widespread attention [1–4]. The presence of human-to-human transmission and large population movements have resulted in the spread of COVID-19 in China, as well as globally, in a short period of time. To reduce socioeconomic losses caused by the pandemic, the Chinese government employed joint prevention and control efforts at the national level and locked down all communities while utilizing the entire country’s manpower, material, and financial resources [5–7]. This ultimately controlled the pandemic in China. To respond to similar public health emergencies in the future, China needs to summarize its experience comprehensively [8, 9] and identify several challenges in its responses to public health emergencies, particularly shortages in public health resources. Such an experience is also beneficial for global pandemic prevention and control.

The global pandemic is becoming increasingly serious, while China’s pandemic has been effectively controlled [10]. As of September 14, 2020, COVID-19 patients were reported in more than 200 countries. The cumulative number of confirmed cases in other countries was 29,099,117, while the cumulative number of deaths was 923,591, suggesting that the situation is spiraling out of control. In the face of the global pandemic, the United States had declared a national state of emergency; the US stock market had hit multiple circuit breakers, a number of companies had declared bankruptcy, and the number of unemployed people had reached 3.28 million by the end of March, which was a historical high. Meanwhile, in the United Kingdom, several senior officials were infected, even as Italy and Spain announced nationwide lockdowns. Although control measures are being continuously strengthened, COVID-19 is still rapidly spreading worldwide.

The World Health Organization (WHO) sent a team to visit China, and its co-lead, Dr. Bruce Aylward, pointed out that the pandemic control measures adapted by China can be considered by other countries. Hence, other countries would not need to start from scratch [11]. The WHO Director-General, Tedros Adhanom, has repeatedly praised China’s pandemic control measures and advocated that various countries should learn from its experience. Among China’s pandemic control measures, such as the addition to medical techniques, national organization, and mobilization, the integration and development of public health resources has particularly attracted attention worldwide. In this context, how do public health resources affect the COVID-19 mortality rate, and how does the severely affected Hubei province in China integrate and enhance public health resources? These questions require significant and appropriate answers that can be considered by other COVID-19-infected countries for the prevention and control of this pandemic. Although Ji et al. (2020) examined the association between the COVID-19 mortality rate and availability of medical resources [12], they did not perform quantitative validation or compile the medical resource integration and development experience of Hubei.

Using data on COVID-19 from the cities in Hubei province in China, this study attempts to fill the gaps in existing research concerning the link between medical resources and the COVID-19 mortality rate. First, we performed empirical analyses with different proxy variables of medical resources, including the number of hospital beds, medical institution beds, and medical staff per confirmed case, to examine the effect of medical resources on the COVID-19 mortality rate. Second, we employed a series of identification strategies including the time-varying effect model and regression discontinuity design to investigate whether the effect varies by time, and empirically examine the effects of policy adjustment and external support on the COVID-19 mortality rate. Finally, we also compiled methods of integration and development of medical resources in China, particularly in the areas that were severely affected by the pandemic.

The contributions of this paper cover the following two areas. First, we enriched the research on COVID-19 pandemic control through our use of linear regression and a time-varying effect model, in addition to a regression discontinuity design, to analyze the effects of medical resources in China on the mortality rate moreover, a normative approach was used to tell the “China’s story.” Second, we collated the methods of integration and development of medical resources in China, particularly in the severely affected areas, and proposed a three-pronged approach to medical resource allocation. This study provides pandemic control recommendations from a medical resource integration perspective to share learnings from China’s experience of pandemic control with the world.

The remainder of this paper is organized as follows. The following section presents the data sources, descriptive statistics and the main empirical methodology. Further, we describe the main results, including the effects of different kinds of medical resources on the COVID-19 mortality rate in Hubei province, the time-varying effect, and the effects of other provincial medical resources on the mortality rate in Hubei province. This is followed by a discussion of the experience of integration and development of medical resources in China, particularly in Hubei province, a severely affected region. The final section concludes the study.

## Methods

### Data and measures

The numbers of confirmed cases of infections and deaths in various cities in Hubei province was obtained from the official website of the Health Commission of Hubei Province [13]. The data were obtained until March 4, 2020. The number of hospital beds, medical staff, and medical institution beds, in addition to traffic mileage and geographical area data of various cities in Hubei province were obtained from the *Statistical Yearbook of Hubei 2018* [14].

The dependent variable for this study was the mortality rate, which was calculated based on the number of deaths and confirmed cases. The number of hospital beds per confirmed patient was the main dependent variable and was calculated from the number of confirmed cases and the total number of hospital beds. Regression specifications adjust for several covariates that may confound estimates of the effect of medical resources on mortality rate, including mortality, number of confirmed cases, traffic density, and whether the city is adjacent to Wuhan (Yes = 1). Traffic density is defined as traffic mileage per square kilometer, which was calculated from traffic mileage and geographical area. The cities neighboring Wuhan are Huangshi, Ezhou, Xiaogan, Huanggan, Xianning, and Xiantao; for the purpose of this analysis, Wuhan is also considered adjacent to Wuhan. Other cities, such as Shiyan, Yichang, Xiangyang, Jingmen, Jingzhou, Suizhou, Enshi, Qianjiang, Tianmen, and Shennongjia, are not adjacent to Wuhan.

### Statistical analysis

Descriptive statistics of the variables are presented in Table 1. The mean mortality rate of various cities in Hubei province as on March 4 was 3.073%, with the highest and lowest mortality rates observed in Wuhan (4.641%) and Shennongjia (0%), respectively. The mean number of hospital beds per confirmed case in various cities was 22.808 beds, with the highest and lowest numbers of hospital beds observed in Xiantao (95.206 beds) and Wuhan (1.845 beds), respectively. The mean number of deaths was 170.706, with the highest and lowest numbers of deaths observed in Wuhan (n = 2,305) and Shennongjia (n = 0), respectively. The mean number of confirmed cases was 3,968.588, with the highest and lowest numbers of confirmed cases observed in Wuhan (n = 49,671) and Shennongjia (n = 11), respectively. The mean traffic density in various cities was 1.546 km, with the highest and lowest densities observed in Ezhou (2.543 km) and Shennongjia (0.286 km), respectively.

**Table 1:** Statistical description of variables.

Variable	N	Mean	S.D.	Min	Max
*mortalityr*	17	3.073	1.25	0	4.641
*perbed*	17	22.808	22.222	1.845	95.206
*mortality*	17	170.706	551.124	0	2305
*infect*	17	3968.588	11812.091	11	49671
*TD*	17	1.546	.47	0.286	2.544
*neighbor*	17	.412	.507	0	1

### Empirical methodology

As the COVID-19 pandemic is almost completely exogenous, an ordinary least squares estimation is employed in this study:

$${mortalityr}_{i}=\alpha_{1}+\beta_{1}*{perbed}_{i}+\delta_{1}X_{i}+\varepsilon_{i}$$ (1)

Here, the subscript *i* refers to the cities in Hubei Province. *mortalityr_i_* represents the mortality rate of the city *i* in Hubei province; *perbed_i_* is the number of hospital beds per confirmed case in the city *i* in Hubei province; Vector *X_i_* denotes the covariates; *α*_1_, *β*_1_, and *δ*_1_ are the estimated parameters; and *ε_i_* is a random error term.

In addition, the time-varying effect model is employed to reexamine the effects on prevention and control of the medical and health resources:

$${mortalityr}_{i}=\alpha_{2}+\beta_{2}*{perbed}_{i}+\beta_{3}*{perbed}_{i}*T+\delta_{2}X_{i}+\varepsilon_{i}$$ (2)

where *T* is the number of days since January 24, 2020. To check the robustness of the results, the following tests were carried out simultaneously:

$${mortalityr}_{i}=\alpha_{3}+\beta_{t}*{perbed}_{i}*T_{t}+\delta_{3}X_{i}+\varepsilon_{i}$$ (3)

Here, *T_t_* is a dummy variable of days t from January 24, 2020.

In addition, in order to analyze the impact of policy adjustment on mortality, a regression discontinuity design was employed to identify the causal relationship between the highest magnitude major public health emergency response of COVID-19 and the mortality rate associated with the disease:

$$\begin{array}{l} LATE=E(y_{1i}-y_{0i}|X=c)=E(y_{1i}|X=c)-E(y_{0i}|X=c)\\ \;=\underset{x↓c}{\lim}{E(y}_{1i}|X)-\underset{x↑c}{\lim}{E(y}_{1i}|X) \end{array}$$ (4)

Here, *LATE* is the local average treatment effect, *y* is the mortality rate, and *c* is the cutoff point. The highest magnitude major public health emergency response in Hubei province was activated at noon on January 24, which is designated as the time cutoff. x is the running variable, which is measured by the number of days before or after January 24. The subscripts 0 and 1 represent before and after the highest magnitude response to the public health emergency was activated, respectively. When employing regression discontinuity, we used a trigonometric kernel function for estimation.

## Results

### Effect of hospital beds on mortality rate

The results of the benchmark regression are presented in Table 2. Column (1) reports the regression results of the mortality rate on the number of hospital beds per capita without covariates. Column (2) reports the estimated results when the number of confirmed cases and death were added as covariates based on Column (1). Column (3) reported the estimated results when traffic density and the variable measuring whether a city is adjacent to Wuhan were added as covariates based on Column (2).

**Table 2:** Number of hospital beds per capita and mortality rate.

	(1)	(2)	(3)
*perbed*	-0.0489**	-0.0518**	-0.0393**
	(0.0190)	(0.0214)	(0.0158)
*mortality*		0.0321	0.0371
		(0.0234)	(0.0239)
*infect*		-0.0015	-0.0017
		(0.0011)	(0.0011)
*TD*			1.299*
			(0.717)
*neighbor*			-0.555
			(0.595)
*_cons*	3.912***	4.360***	2.413*
	(0.307)	(0.481)	(1.157)
*N*	17	17	17
*R*^*2*^	0.411	0.514	0.645

Note

***, **, and * indicate significance at 1%, 5%, and 10%, respectively. Robust standard errors are presented in parentheses.

As presented in Table 2, the estimated coefficients for the number of hospital beds per capita are negative and significantly different from zero, suggesting that the number of hospital beds per capita has a significant negative effect on the mortality rate. This implies that increasing the number of hospital beds per capita will decrease the mortality rate. Specifically, according to Column (3), an increase in the number of hospital beds per capita by 10 will result in a decrease in the COVID-19 mortality rate by 0.393%. The main reason is that hospitals are overloaded during the COVID-19 pandemic, and obtaining a bed translates to an opportunity for patient treatment.

### Effect of medical resources on mortality rate

#### Effect of number of medical institution beds on mortality rate

As presented in Table 3, the number of medical institution beds per capita also has a significant negative effect on the COVID-19 mortality rate. Specifically, increasing the number of medical institution beds per capita by 10 will decrease the mortality rate by 0.3%, which is slightly lower than the pandemic control effect of the number of hospital beds per capita. The main reasons are as follows. First, increasing the number of nonhospital beds will solve the patient-bed conflict and aid in pandemic control. Second, there should be a suitable ratio between the number of beds and the number of medical staff. If only the number of beds is increased but not the number of medical staff, there will be positive effects on pandemic control, but the contribution would be low. Therefore, the number of medical staff should also be increased when increasing the number of hospital beds to effectively control the pandemic.

**Table 3:** Number of medical institution beds per capita and mortality rate.

	(1)	(2)	(3)
*perbed*	-0.0312**	-0.0315**	-0.0231*
	(0.0143)	(0.0144)	(0.0108)
*mortality*		0.0259	0.0355
		(0.0281)	(0.0245)
*infect*		-0.0012	-0.0016
		(0.0013)	(0.0011)
*TD*			1.560*
			(0.850)
*neighbor*			-0.554
			(0.607)
*_cons*	3.784***	4.084***	1.833
	(0.298)	(0.589)	(1.370)
*N*	17	17	17
*R*^*2*^	0.307	0.396	0.604

Note

***, **, and * indicate significance at 1%, 5%, and 10%, respectively. Robust standard errors are presented in parentheses.

#### Effect of the number of medical staff on the mortality rate

In Table 4, the coefficient of the number of medical staff per capita is significantly negative, indicating that increasing the number of medical staff per capita can significantly reduce the mortality rate. According to Column (3) in Table 4, an increase in the number of medical staff per capita by 10 will decrease the mortality rate by 0.24%, which is lower than the control effect of the number of hospital beds. This is because there is a severe shortage of medical staff during the pandemic, and hence, the hospitals and medical staff are overburdened. According to official reports, the fever outpatient department of Wuhan Union Hospital examined 800 people every day at the peak of the pandemic, but was still unable to satisfy the diagnosis and treatment needs of these patients. Considering that one medical staff can examine several patients, but one bed can only satisfy the hospitalization requirement of one patient, the control effect of the medical staff is, therefore, lower than that of the number of hospital beds.

**Table 4:** Number of medical staff per capita and mortality rate.

	(1)	(2)	(3)
*perstaff*	-0.0242**	-0.0250**	-0.0183*
	(0.0106)	(0.0102)	(0.0091)
*mortality*		0.0274	0.0373
		(0.0298)	(0.0256)
*infect*		-0.0013	-0.0017
		(0.0014)	(0.0012)
*TD*			1.633*
			(0.870)
*neighbor*			-0.575
			(0.655)
*_cons*	3.786***	4.121***	1.766
	(0.298)	(0.631)	(1.404)
*N*	17	17	17
*R*^*2*^	0.258	0.352	0.585

Note

***, **, and * indicate significance at 1%, 5%, and 10%, respectively. Robust standard errors are presented in parentheses.

### Effects of medical resource changes on mortality rate

In contrast to the timepoint static analysis performed in the previous sections, we now analyze the effects of policy adjustment and external support on the COVID-19 mortality rate in this section. First, the time-varying effect model was used to dynamically examine the control effect of medical resources. Second, the regression discontinuity design was employed to analyze the effects of the highest magnitude response policies for public health emergencies in Hubei province on the mortality rate. Third, linear regression was used to examine the effect of support provided to Hubei province by the other cities in China on the mortality rate in Hubei province.

#### Time-varying effect of mortality rate under medical resource integration

Table 5 provides the estimated results of the time-varying effect of the mortality rate on the number of hospital beds per confirmed case. According to Columns (2) and (3) in Table 5, the main independent variable was changed to the interaction terms between the number of hospital beds per capita and dummy variables indicating different period; Column (2) reports the estimated results when an interval of 3 days was used, while Column (3) reports the estimated results when an interval of 5 days was used. The results of the time-varying effect estimation are still significantly negative. This suggests that the mortality rate will decrease with the integration and development of medical resources as the pandemic develops.

**Table 5:** Medical resource integration and development effects.

	(1)	(2)	(3)
*perbed*	-0.0002*		
	(0.0001)		
perbed*T	0.0001		
	(0.0001)		
perbed*T_*1*_		-0.0002**	-0.0002**
		(0.0001)	(0.0001)
perbed*T_*4*_		-0.0024	
		(0.0044)	
perbed*T_*6*_			-0.0053
			(0.0088)
perbed*T7		-0.0079	
		(0.0107)	
perbed*T_*10*_		-0.0305***	
		(0.0097)	
perbed*T_*11*_			-0.0328***
			(0.0107)
perbed*T_*13*_		-0.0446***	
		(0.0132)	
perbed*T_*16*_		-0.0550***	-0.0524***
		(0.0149)	(0.0139)
perbed*T_*19*_		-0.0454**	
		(0.0185)	
perbed*T_*21*_			-0.0485***
			(0.0183)
perbed*T_*22*_		-0.0533***	
		(0.0187)	
perbed*T_*25*_		-0.0532***	
		(0.0181)	
perbed*T_*26*_			-0.0509***
			(0.0159)
perbed*T_*28*_		-0.0487***	
		(0.0168)	
perbed*T_*31*_		-0.0459**	-0.0423**
		(0.0184)	(0.0174)
perbed*T_*34*_		-0.0430**	
		(0.0182)	
perbed*T_*36*_			-0.0365**
			(0.0169)
perbed*T_*37*_		-0.0396**	
		(0.0178)	
perbed*T_*41*_		-0.0341*	-0.0305*
		(0.0185)	(0.0177)
*_cons*	2.779***	3.441***	3.327***
	(0.101)	(0.442)	(0.409)
*covariates*	Yes	Yes	Yes
*N*	706	770	770
*R*^2^	0.080	0.004	0.002

Note

***, **, and * indicate significance at 1%, 5%, and 10%, respectively. Robust standard errors are presented in parentheses.

#### Major public health emergency magnitude response effects

The estimation results presented in Table 6 show that major public health emergency magnitude responses can decrease the mortality rate during the pandemic, but its effects are insignificant. This is attributed to the following reasons. First, the highest magnitude major public health emergency response is an integrated policy. Second, there is a time lag in policy effects (i.e., medical resources continuously increase after policies are implemented). Third, the mortality rate changes as well to conform to epidemiological stable recovery patterns in addition to the effects of policies.

**Table 6:** Highest magnitude response policy effects for the major public health emergency.

	(1)	(2)	(3)	(4)
*Bandwidth*	-/+ 10 days	-/+ 5 days	-/+ 3 days	-/+ 1 days
*LATE*	-8.651	-9.515	-11.09	-11.79
	(7.800)	(9.369)	(11.90)	(14.09)
*N*	259	174	140	107

Note

***, **, and * indicate significance at 1%, 5%, and 10%, respectively. Robust standard errors are presented in parentheses.

#### Effects of external medical personnel supporting Hubei

To evaluate the effect of the external assisting medical staff on Hubei’s pandemic treatment work, we used the number of these external medical staff per confirmed case as the main independent variable for reevaluation. As shown in Table 7, the estimation results demonstrate that the number of assisting medical staff per capita significantly decreased the mortality rate in the pandemic region. Every increase in the number of assisting medical staff per capita by 10 decreases the mortality rate by 0.134%. Up until March 4, the number of assisting medical teams from the People’s Liberation Army and various provinces was 42481. These assisting medical staff improved treatment capabilities, thereby decreasing the mortality rate.

**Table 7:** Number of assistance medical staff per capita and mortality rate.

	(1)	(2)	(3)
*persist*	-0.0154***	-0.0175***	-0.0134**
	(0.0012)	(0.0021)	(0.0047)
*die*		0.0373	0.0419
		(0.0298)	(0.0287)
*infect*		-0.0017	-0.0019
		(0.0014)	(0.0013)
*TD*			0.652
			(0.645)
*neighbor*			0.174
			(0.500)
*_cons*	3.303***	3.754***	2.698**
	(0.260)	(0.460)	(1.160)
*N*	17	17	17
*R*^2^	0.400	0.578	0.625

Note

***, **, and * indicate significance at 1%, 5%, and 10%, respectively. Robust standard errors are presented in parentheses.

## Discussion

Based on our analyses, increasing medical resources will decrease the mortality rate during a pandemic. Therefore, a question arises as to how medical resources can be integrated and developed during a pandemic. Based on the pandemic control experience of Hubei province, we recommend a three-pronged approach. First, medical resources within the hospital are integrated by removing barriers among the various departments. Subsequently, the resources of regional healthcare systems are integrated. Finally, the external medical resources of the healthcare system are developed and used.

### Integration of medical resources within the hospital

According to current epidemiological surveys, the clinical presentations of COVID-19 are mainly fever, fatigue, and dry cough. Therefore, the infectious disease department, respiratory medicine and critical care medicine department, emergency department, intensive care unit, and fever outpatient departments are usually the frontline departments responsible for pandemic control and for responding to the constraints in medical staff, beds, and medical supplies. On the contrary, other hospital departments are less affected by the pandemic. Additionally, due to concentrated outbreaks, it is considered inappropriate when only some of the hospital departments will be responsible for the control and prevention of this pandemic. Therefore, it would be more effective to remove the barriers among different departments and pool together the medical staff, beds, and medical supplies from various departments for treating COVID-19 patients.

#### Overcoming departmental barriers and interdepartmental bed allocation

During the pandemic, the Union Hospital of Tongji Medical College of Huazhong University of Science and Technology was recognized as being at the “frontline of China’s war against the pandemic.” In addition to the main hospital and the west branch that is involved in treating COVID-19 patients, the tumor center was also used to treat COVID-19 patients. Therefore, a large proportion of medical resources was used in pandemic control. Under the premise of ensuring medical safety, treatment beds that were dispersed across various departments were brought together for allocation, with priority provided to the critical care department to treat severe patients [15].

#### Drawing and integrating medical staff from various departments

According to statistics, the area of the original fever outpatient department in Wuhan Union Hospital was expanded by five times after the pandemic occurred. On average, the fever outpatient department 400 patients a day; 10 physicians were arranged for daily consultation, and a “three-shift” system was implemented. The expansion of the fever outpatient department, an increase in the number of consultation patients, and the resultant increase in waiting time would require more frontline medical staff. Medical staff in the hospital were drawn and integrated to support the fever outpatient department, and they were flexibly deployed based on the fluctuations in consultations to effectively alleviate medical staff constraints. For example, more than 200 medical staff from 20 departments in Wuhan Union Hospital are at the frontline of this pandemic, and additional staff were added based on the variations in the pandemic. The Qingdao Municipal People’s Hospital drew 16 workers from administrative departments for prescreening and triage work to alleviate the long-term pressure experienced by the frontline medical staff.

### Integration of medical resources in the healthcare system

The regional healthcare system includes hospitals, grassroots medical institutions, public health institutions, and other medical institutions. According to statistics, there were 6,340 medical institutions in Wuhan in 2018, which included 398 hospitals, 5,853 grassroots medical institutions, and 79 professional public health institutions. The total number of beds was 95,900, and medical resources were abundant. Due to the differences in the number of infected people in the region—for example, more infected people are observed near the “origin” of the infection—irrational consultation behavior such as scrambling to major hospitals is observed. The treatment pressure on the healthcare system differs by region, resulting in a supply-demand mismatch. Therefore, the rational deployment of medical resources in a regional healthcare system is extremely necessary during the pandemic.

There were 61 tertiary hospitals, 64 secondary hospitals, 164 primary hospitals, and 109 unclassified hospitals in Wuhan. When the COVID-19 outbreak emerged, patients usually selected renowned tertiary hospitals with strong integration or strengths in related diseases. This resulted in high consultation pressure in tertiary hospitals and low consultation pressure in less popular hospitals or lower-level hospitals. The massive influx of patients to major hospitals increased not only the job stress of medical staff in these hospitals but also the long queues for consultation, increasing the possibility of cross-infection. To prevent patients from seeking medical attention blindly, Wuhan set up 61 fever outpatient departments, of which 41 were located in the downtown area, 20 were located in the new city area, 16 were municipal hospitals, and 9 were designated medical institutions. Patients were asked to seek medical attention at nearby medical institutions, and regional medical institutions were fully utilized for the prescreening and triage of fever patients.

With respect to medical staff, there were a total of 136,300 medical staff employed in medical institutions in Wuhan, of which 109,600 were medical technologists, 39,600 were practicing (assistant) physicians, 54,400 were registered nurses, 4,700 were pharmacists, and 5400 were technicians. To date, there have been more than 20,000 COVID-19 patients who are receiving inpatient treatment in Wuhan. In particular, it is impossible for the medical staff of designated tertiary hospitals to manage the huge consultation and treatment work by themselves. Hence, the medical staff can be effectively utilized by rationally dispatching medical staff from medical institutions within the city to support designated tertiary hospitals so that the number of patients is proportionate to the number of medical staff.

In terms of the number of beds in Wuhan’s medical institutions, there were 81,700, 6,400, and 4,500 beds in hospitals, medical service institutions, and health centers, respectively. While Wuhan hospitals have a sufficient number of beds, after subtracting the beds used for other diseases and confirmed COVID-19 patients, the number of beds available for quarantine and observation of suspected COVID-19 patients was found to be extremely limited. Considering this, beds from medical service institutions and health centers, approximately 10,000 beds, can be used to alleviate short-term bed shortages. However, other measures need to be adapted to address long-term bed shortages.

### Development of medical resources

#### Opening new hospitals and increasing bed supply

As the number of COVID-19-infected people continuously increases, the existing number of beds cannot satisfy the hospitalization requirements of the patient, and new hospitals should be constructed as soon as possible to increase bed supply. In 2003, a severe acute respiratory syndrome (SARS) outbreak occurred. During the peak of the pandemic, the Xiaotangshan Hospital in Beijing was constructed within 7 days. The hospital has an area of 25,000 m^2^ and can hold 1,000 beds. It treated one-seventh of China’s SARS patients within two months, and its construction was considered to be a turning point in the pandemic control of SARS. Currently, a severe bed shortage is also encountered due to the COVID-19 pandemic. Using the experience of Xiaotangshan Hospital, Wuhan spent ten days to construct the Huoshenshan Hospital, followed by the Leishenshan Hospital. While the former has 1,000 beds, the latter has 1,600. Additionally, 11 cabin hospitals were constructed to provide more than 10,000 isolation beds for suspected or mild COVID-19 patients. The construction of these temporary hospitals significantly alleviated the problems of bed shortage and the lack of treatment for patients. Not only were temporary hospitals constructed in the worst-affected city of Wuhan, but they were also successively built in other regions. Huanggang in Hubei province urgently converted the Dabieshan Medical Center into an isolation center with more than 1,000 beds. Xi’an in Shaanxi constructed an emergency zone in a public health center to provide 500 beds. Qibuoshan Hospital in Zhengzhou in Henan province provided 800 beds, while Luoyang constructed an emergency treatment center with 304 beds. The role of temporary hospitals in increasing bed supply to treat inpatients cannot be ignored.

#### Alleviating pressures on frontline staff and dispatching medical staff from various provinces

To mitigate the burden on the frontline medical staff, on one hand, internal deployment adjustment between various departments in the hospital and within the healthcare system can be performed. On the other hand, staff can be deployed from regions experiencing a milder COVID-19 pandemic. For example, during this pandemic, the People’s Liberation Army medical team and those from other provinces provided emergency assistance for Hubei province, and 32,000 medical staff from other provinces became an important part of the task force for COVID-19 pandemic control in Hubei province. This included the first batch of 150 medical staff for Hebei province and 10 staff members in the second batch of the epidemiological survey team. The First Affiliated Hospital of the School of Medicine of Zhejiang University and Sir Run Shaw Hospital each sent 137 medical staff to severely affected regions in Wuhan. A total of 122 traditional Chinese medical staff from Shanghai were sent to Leishenshan Hospital in Wuhan. Simultaneously, to improve the results of such assistance, China has adopted a one-to-one precise assistance method so that various provinces can be matched to various cities in Hubei province, with a clear understanding of responsibilities and proper implementation, thereby ensuring that the medical needs of various cities are satisfied.

#### A joint effort by different segments of society to support medical supplies

During the pandemic, the demand for masks, protective gowns, surgical clothes, alcohol, and other protective supplies significantly increased, with several hospitals in China seeking donations from the public. This was compounded by a delayed resumption of work due to the Chinese New Year in China, which caused a widespread shortage of equipment, personnel, and protective gear in medical institutions. Relevant government agencies in China actively advocated and guided companies and charitable organizations to support protective gear, medical devices, and drugs and provide timely assistance to Hubei province. Simultaneously, joint pandemic control mechanisms were fully utilized, resumption of work and manufacturing were promoted in key companies, production was expanded, and unified deployment of key pandemic control supplies was implemented to ensure a sufficient medical supplies. The management of medical institutions followed the principle of focused coordination, professional management, guaranteeing urgent needs, and dedicated materials for dedicated use, and the number of department reserves was clarified, and fixed amount requisitioning and periodic inventory taking was performed.

## Conclusion

Hubei province in China was severely affected in the COVID-19 pandemic. The aim of the this study was is to investigate the association of medical resources with the mortality rate of patients in Hubei province and to summarize the experience and implications in pandemic prevention and control from the perspective of medical resource integration. We examined the impact of medical resources on the local mortality rate and the manner in which Hubei province integrated and developed their medical resources, improving their treatment capacity and efficiency during the pandemic.

First, a linear regression was used for statistical analysis, and the results showed that the numbers of hospital beds, medical institution beds, and medical staff per capita had significant negative effects on COVID-19 mortality. Increasing the number of hospital beds per capita by 10, the number of medical institution beds per capita by 10, and the number of medical staff per capita by 10 decreased the mortality rates by 0.393%, 0.3%, and 0.24%, respectively. Second, a time-varying effect model and regression discontinuity design were used for the dynamic investigation of the effects of medical resources on mortality rate, and the following results were obtained. First, the mortality rate decreased with the integration and development of medical resources. Second, primary medical responses decreased the COVID-19 mortality rate. Third, the greater the number of external staff assisting in the pandemic regions, the lower the mortality rate. Our analysis, combined with the pandemic control experience of Hubei province, has implications for global pandemic prevention and control. We propose that medical resources within the hospitals should be integrated by breaking the barriers among various departments. This should be followed by integration of resources within the regional healthcare system, subsequently leading to strengthening of the external resources of the healthcare system. Overall, Our findings can provide insights on pandemic control for the rest of the world, where the situation has been consistently deteriorating and has been more severe than that in China.

## Supporting information

S1 Dataset (XLSX)
